# Gonadectomy reduces the density of androgen receptor-immunoreactive neurons in male rat’s hippocampus: testosterone replacement compensates it

**DOI:** 10.1186/s12993-016-0089-9

**Published:** 2016-01-28

**Authors:** Sajjad Moghadami, Mehrdad Jahanshahi, Hamid Sepehri, Hossein Amini

**Affiliations:** 1 Neuroscience Research Center, Department of Anatomy, Faculty of Medicine, Golestan University of Medical Sciences, Km 4 Gorgan-Sari Road (Shastcola), P.O. Box 4934174515, Gorgan, Iran; 2 Neuroscience Research Center, Department of Physiology, Faculty of Medicine, Golestan University of Medical Sciences, Gorgan, Iran; 3 Neuroscience Research Center, Department of Pharmacology, Faculty of Medicine, Golestan University of Medical Sciences, Gorgan, Iran

**Keywords:** Testosterone, Gonadectomy, Spatial memory, Androgen receptor, Hippocampus

## Abstract

**Background:**

In the present study, the role of gonadectomy on memory impairment and the density of androgen receptor-immunoreactive neurons in rats’ hippocampus as well as the ability of testosterone to compensate of memory and the density of androgen receptors in the hippocampus was evaluated.

**Methods:**

Adult male rats (except intact-no testosterone group) were bilaterally castrated, and behavioral tests performed 2 weeks later. Animals bilaterally cannulated into lateral ventricles and then received testosterone (10, 40 and 120 µg/0.5 µl DMSO) or vehicle (DMSO; 0.5 µl) for gonadectomized-vehicle group, 30 min before training in water maze test. The androgen receptor-immunoreactive neurons were detected by immunohistochemical technique in the hippocampal areas.

**Results:**

In the gonadectomized male rats, a memory deficit was found in Morris water maze test on test day (5th day) after DMSO administration. Gonadectomy decreased density of androgen receptor-immunoreactive neurons in the rats’ hippocampus. The treatment with testosterone daily for 5 days attenuated memory deficits induced by gonadectomy. Testosterone also significantly increased the density of androgen receptor-immunoreactive neurons in the hippocampal areas. The intermediate dose of this hormone (40 µg) appeared to have a significant effect on spatial memory and the density of androgen receptor-immunoreactive neurons in gonadectomized rats’ hippocampus.

**Conclusions:**

The present study suggests that testosterone can compensate memory failure in gonadectomized rats. Also testosterone replacement can compensate the reduction of androgen receptor-immunoreactive neurons density in the rats’ hippocampus after gonadectomy.

## Background

Hippocampus, an important subcortical structure in the mammalian brain, has been shown to be involved in several types of learning and memory processes [1–3]. In most mammals, the hippocampus has a well-documented role in spatial memory acquisition [4]. Hippocampus is also known as a target for different neuroactive steroids such as androgens [5–9] and also this hormone plays a fundamental role for the regulation of the structure and function of the hippocampus [10–14].

Androgens may influence cognitive performance [15]. Some reports showed that androgens are involved in the modulation of learning and memory [15, 16]. Changes in gonadal androgen levels over the time of life, in addition to cause the variation in cognitive function, make some type of neurodegenerative disorder such as Alzheimer diseases (AD) [16–21]. However, the literature describing the effects of androgens on spatial memory is complex and contradictory [22–24]. Evidence for both positive and negative correlations can be found [3]. Some evidence suggests that androgens can impair memory in animals [25]. Such beneficial neural actions of androgens include: (1) promotion of neuron growth, axonal regeneration, and synaptic function, (2) protection against neuronal cell loss, and (3) regulation of AD-related pathology, including amyloid β accumulation. In the first case, androgens have a variety of growth-promoting and maturational effects on neurons in a range of paradigms [26–29]. In addition, androgens are potent facilitators of spine density and synaptic function, including regulation of long-term potentiation in the hippocampus [30–34]. Androgens also accelerate regeneration of axons in damaged motor neurons in certain experimental paradigms [35–38]. Pharmacological and genetic tools indicate that androgen receptor activation contributes to these actions [31, 32].

In the brain, androgen receptors are expressed by both neurons and glial cells and are mainly found in the thalamus, hypothalamus, hippocampus, amygdala and cerebral cortex [5, 39, 40]. In the rat hippocampus, androgen receptor is particularly concentrated in CA1 pyramidal cells [41, 42]. It is reasonable to assume the presence of a relationship between androgen receptors and cognitive activity [24, 43, 44]. Males have higher levels of circulating androgens than females, and androgens often regulate the expression of their own receptors [45, 46], based on these reports. Thus, the aim of this study was to investigate the role of gonadectomy and testosterone replacement on memory and the density of hippocampal androgen receptor-immunoreactive (ir) neurons.

## Methods

### Animals

Adult male Wistar rats (200 ± 220 g) were obtained from the Pasteur Institute (Tehran, Iran). They were housed in groups of five in the big cage before surgery and individually in small cages after surgery. The animals were kept at a constant temperature (22 ± 2 °C) under a 12 h/12 h light–dark cycle (light beginning at 07:00 a.m.) and they had free access to food and water. All experiments were carried out during the light phase and all procedures described herein were approved by the Ethic Committee of Golestan University of Medical Sciences, Iran. All efforts were made to minimize the number of animals and their suffering.

### Gonadectomy

The animals in all groups, except the intact-no T group, were bilaterally castrated to reduce circulating levels of testicular secretions. The rats were anesthetized with a mixture of ketamine (100 mg/kg, i.p.) and xylazine (10 mg/kg, i.p.). After the onset of anesthesia, gonadectomy was performed through a ventral incision in the scrotum [23]. Animals were allowed to recover for at least 14 days.

### Intracerebroventricular (ICV) cannula surgery

Rats were anesthetized with a ketamine/xylazine mixture and placed in a stereotaxic frame (David Kopf Instruments, USA) with flat-skull position. Under sterile conditions, rats were implanted with a 21-gauge guide cannulae in the right and left lateral ventricles, at the following coordinates with reference to Bregma: anterior/posterior −0.8 mm; medial/lateral ± 1.5 mm; dorsal/ventral −4.2 mm [47]. The bilateral guide cannulae were attached to the skull surface using dental cement and jeweler’s screws and then stainless steel stylets (27 gauge) were inserted into the guide cannulae to maintain patency prior to microinfusions. Animals were allowed to recover for at least 7 days.

### ICV injections

For bilateral drug infusion, the animals were gently restrained by hand; the stylets were removed from the guide cannulae and replaced by 27-gauge injection needles connected to polyethylene tubing to 10 µl Hamilton micro-syringes. The injection needle was inserted 0.5 mm beyond the tip of guide cannulae. The injection solutions were administered in a total volume of 1 μl/rat (0.5 μl in each side of lateral ventricles) over a 60 s period. Injection needles were left in place for an additional 60 s to facilitate the diffusion of the drugs. ICV Injections were done 30 min before behavioral testing in five consecutive days.

### Experimental design

Following a 1-week recovery from cannula surgery, rats were randomly assigned to one of the following five groups (n = 6):

- *Intact*-*no testosterone treatment (no T) group* without gonadectomy (GDX) and cannula surgery, no drug treatment;
- *GDX*-*vehicle group* with GDX and cannula surgery, receiving bilateral microinjection of 0.5 μl vehicle (DMSO; dimethyl sulfoxide, Merck, Germany);
- *Three GDX*-*testosterone treated groups* with GDX and cannula surgery, receiving bilateral microinjection of testosterone (Sigma, USA) with different doses (10, 40, and 120 μg/0.5 μl).

Testosterone was dissolved in DMSO and in all experiments, DMSO was used as a vehicle that has no significant effect on learning and memory in the Morris water maze [44].

### Spatial learning and memory task

Spatial learning and memory (reference memory testing) were investigated in intact-no T, GDX-vehicle and GDX-T treated rats using the Morris water maze paradigm. As we have explained this technique in the previous studies [24, 48, 49], the rats were trained in a circular swimming pool (140 cm in diameter and 50 cm deep). The pool was placed in a room surrounded by constant visual cues on curtains around the pool and filled with water (24 °C) to a depth of 35 cm. The water maze was divided virtually into four equal quadrants (north, south, east, and west). In the middle of the north quadrant, a platform (10 cm in diameter) was placed at 1 cm beneath the surface of the water.

Four training trials were conducted each day for four consecutive days. Each trial began from one of the four starting positions which are changed across days. Starting positions were varied in a quasi-random fashion so that in each block the subject started from each location once and never started from the same place. During each trial, the rat was placed in the water facing the wall of the maze and given 60 s to locate the platform. It was allowed to remain on the platform for 20 s, and was then removed. If a rat failed to locate the platform, it was gently guided and allowed 20 s on top of the platform. After the final trial, the rats were toweled dried and placed in a holding cage under a heating lamp before being returned to the home cage.

On the test day (5th day) following the last day of training, the animals underwent a 60 s probe trial to determine the extent to which they had learned the location of the platform.

The routs, used by rats, were recorded by infra-red digital camera connected to a tracking system software (mice router, Urimia Instrument Inc., Iran) were mounted above the pool to allow thorough analysis of each trial and calculate the latency to find the hidden platform, swim path length and swimming speed.

### Tissue preparation for immunohistochemistry

Forty-eight hours after spatial memory evaluation, the rats were deeply anesthetized with chloroform. Then, the rats were transcardially perfused with 0.9 % saline, followed by 4 % paraformaldehyde (Merck, Germany) freshly prepared in phosphate sodium buffer (PBS; Gibco, UK). The brain tissue was isolated and fixed in 4 % paraformaldehyde for 1 week. Different degrees of alcohol were used for dehydration, followed by clarification in xylene. After histological processing, the brain tissue was impregnated and then embedded in paraffin wax. Finally, serial coronal sections were collected with 6-μm thickness by a rotary microtome (Pooyan MK 1110, Iran) from bregma −2.40 to −4.56 mm of the hippocampal formation [47]. An interval of 20 μm was placed between each two consecutive sections.

### Immunohistochemistry

For immunohistochemistry, 6 rat brains were cut in each group and six slices from anterior to posterior of hippocampus were selected. Slices were stained with antibody against androgen receptor. The sections were first incubated at 37 °C for 30 min and then embedded in xylene for deparaffinization and hydrated through a graded series of ethanol and finally rinsed with distilled water. The sections were incubated at 60 °C for 5 min. Following this stage the sections were covered with epitope retrieval solution (IHC World, USA) at 90 °C for 15 min and allowed them to cool for 20 min at room temperature and rinsed with washing buffer (PBS/Tween 20 in 0.1 % Triton X-100). Afterwards, the block step was carried out with a peroxidase blocking solution (IHC World, USA) for 10 min at room temperature and then rinsed with washing buffer. Next, the sections were blocked with avidin/biotin blocking solution (IHC World, USA) for 30 min at room temperature and rinsed with PBS. Afterwards, the sections were incubated with the Anti-Androgen Receptor Rabbit monoclonal antibody (1:250, Abcam Inc., USA) at 37 °C for 60 min and rinsed with washing buffer. Then, sections were incubated with biotinylated goat anti rabbit IgG (Abcam Inc., USA) at 37 °C for 60 min and rinsed with washing buffer. These sections were incubated with Streptavidin HRP protein (1:5000, Abcam Inc., USA) for 30 min at room temperature and rinsed with washing buffer. The sections were visualized by using DAB (Dako, Denmark) and counterstained lightly with Meyer’s hematoxylin. Finally, the sections were dehydrated in ethanol, cleared in xylene and coverslipped with entellan (Merck, Germany).

### Image processing and cell counting

All Images were recorded by using a digital camera (DP 72, Olympus, Japan) equipped with a light microscope (BX51, Olympus, Japan) under a magnification of 400 for CA1 and CA3 areas and magnification of 1000 for dentate gyrus (DG) area. A field of 30,000 µm^2^ for CA1 and CA3 areas and also 4800 µm^2^ in DG area was selected randomly in the all sections. To measure the area density of the androgen receptor-ir neurons, using OLYSIA Autobioreport software (Olympus, Japan), the appropriate grids were superimposed on the pictures, and the cells were counted manually [50, 51].

### Data analysis

All data were statistically analyzed using SPSS v.16 (Armonk, NY, USA). Statistical evaluation was carried out by Kolmogorov–Smirnov test to examine the normal distribution. Data were analyzed by one-way analysis of variance (ANOVA) followed by post hoc least significance difference (LSD) test for over-all multiple comparisons. For all comparisons, P < 0.05 was considered significant.

## Results

### Behavioral results

**Fig. 1:**
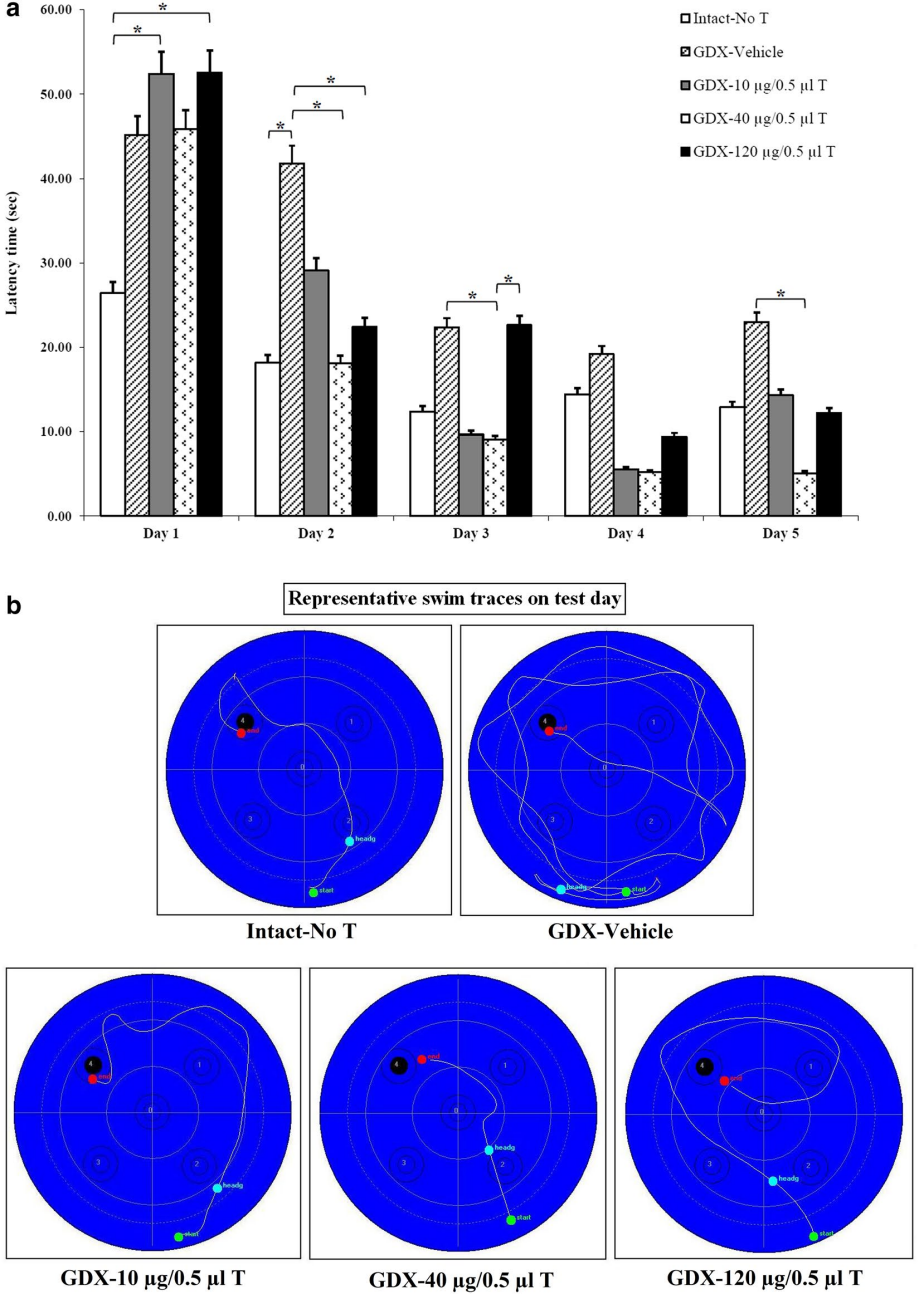
Effect of ICV administration of testosterone and DMSO on acquisition of spatial memory in Morris water maze task. **a** Comparison latency time to reach hidden platform. The figure shows that gonadectomy (GDX-vehicle group) caused memory impairments via increasing latency on test day. The rats receiving 40 µg/0.5 µl dose of testosterone showed significant decrease in latency at compared to the GDX-vehicle group on test day. Data are presents as mean. *P ≤ 0.05 means significant. **b** Representative swim traces in Morris water maze test

A 5-day training program designed for each ICV injection of testosterone (Fig. 1). Considering the same training days in different groups, the results showed that gonadectomy (GDX-vehicle group) caused memory impairments via increasing latency on test day. The rats receiving 40 µg/0.5 µl dose of testosterone showed significant decrease in latency at compared to the GDX-vehicle group (P < 0.05) on test day. However, low and high doses of testosterone (10 and 120 µg/0.5 µl) had no significant differences compared to the GDX-vehicle group, in latency parameter (P > 0.05).

**Fig. 2:**
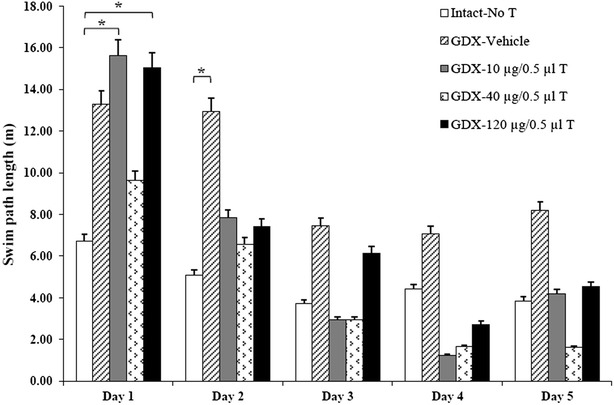
Comparison swims the path length in Morris water maze test. The gonadectomy increased to swim path length in comparison with the intact-no T group on test day. Administration of various doses of testosterone decreased swim path length in comparison with the GDX-vehicle group on test day. Data are presents as mean. *P ≤ 0.05 means significant

The gonadectomy increased to swim path length in comparison with the intact-no T group on test day (Fig. 2). Administration of various doses of testosterone decreased swim path length in comparison with the GDX-vehicle group on test day. High decrease in this parameter observed in testosterone-treated group (40 µg/0.5 µl), but this decrease was not statistically significant.

**Fig. 3:**
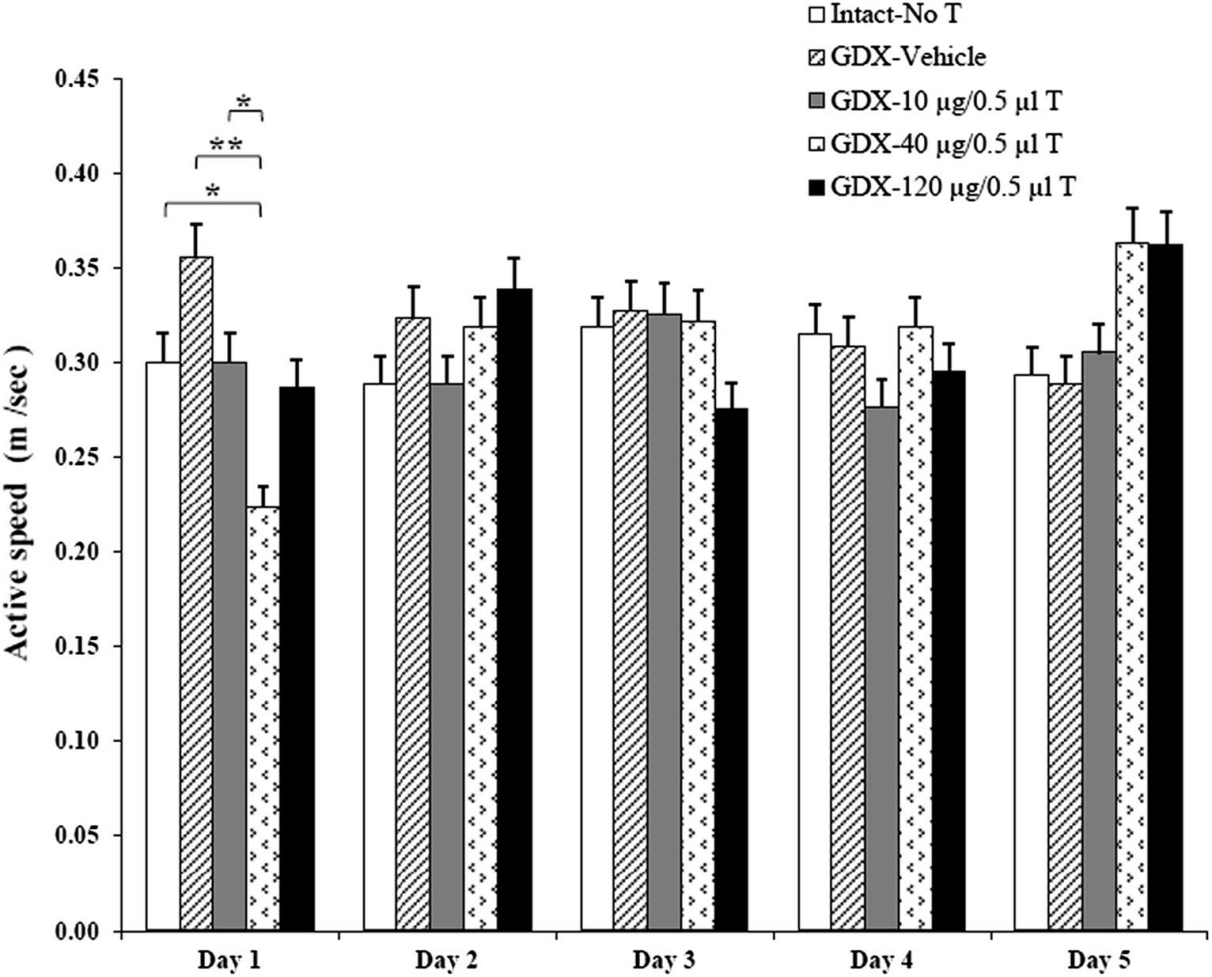
Comparison active speeds in Morris water maze test. There was no significant difference in swimming speed between all studied groups on test day. Data are presents as mean. *P ≤ 0.05; **P ≤ 0.01 means significant

There was no significant difference in swimming speed between all studied groups on test day (Fig. 3). These results indicated that injection of DMSO and testosterone had no effect on swimming speed in the Morris water maze task.

### Histological results

**Fig. 4:**
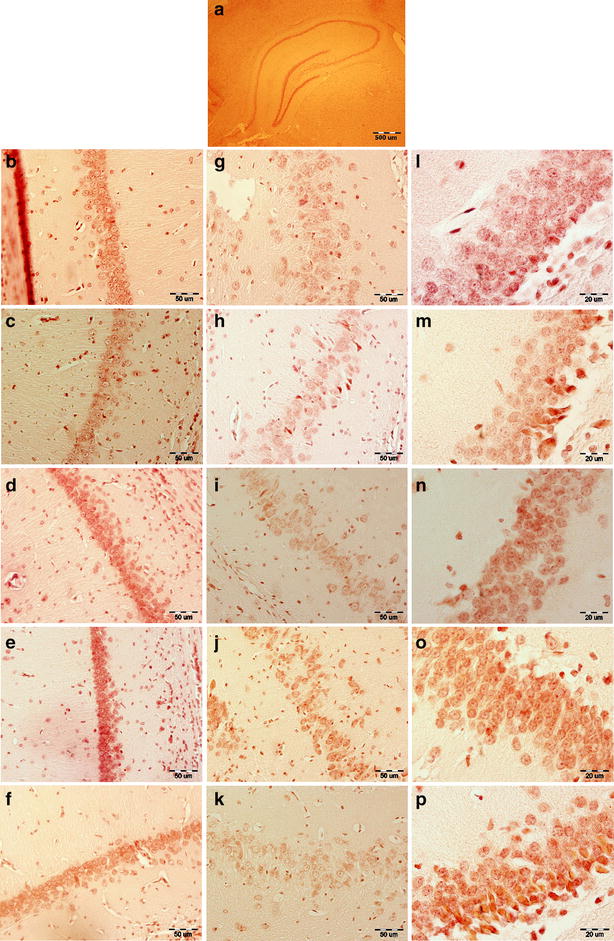
Coronal sections of rat hippocampus stained by immunohistochemistry with an antibody against androgen receptor. **a** Androgen receptor-ir neurons in the whole hippocampus. **b–f** Androgen receptor-ir neurons in the CA1 area of hippocampus at intact-no T (**b**), GDX-vehicle (**c**), GDX-10 µg/0.5 µl T (**d**), GDX-40 µg/0.5 µl T (**e**) and GDX-120 µg/0.5 µl T (**f**) groups. **g–k** Androgen receptor-ir neurons in the CA3 area of hippocampus at intact-no T (**g**), GDX-vehicle (**h**), GDX-10 µg/0.5 µl T (**i**), GDX-40 µg/0.5 µl T (**j**) and GDX-120 µg/0.5 µl T (**k**) groups. **l–p** Androgen receptor-ir neurons in the DG area of hippocampus at intact-no T (**l**), GDX-vehicle (**m**), GDX-10 µg/0.5 µl T (**n**), GDX-40 µg/0.5 µl T (**o**) and GDX-120 µg/0.5 µl T (**p**) groups. *Scale bars* show 50 μm for **b**–**f** and **g**–**k**, and 20 μm for **l**–**p**

Figure 4 shows density of androgen receptor-ir neurons in the male rat hippocampus. It is shown that, gonadectomy decreased the number of androgen receptor-ir neurons in hippocampal CA1, CA3, DG areas (Table 1). The decrease in the number of androgen receptor-ir neurons was significantly different only in the CA1 area between GDX-vehicle and intact-No T groups (P < 0.001), F = 71.89.

**Table 1:** The number of androgen receptor-ir neurons in male rat hippocampus (mean ± SD)

Areas	CA_1_ (30,000 μm^2^)		CA_3_ (30,000 μm^2^)		DG (4800 μm^2^)	
Groups	Mean ± SD	P value	Mean ± SD	P value	Mean ± SD	P value
Intact-no T	21.92 ± 2.911	0.000***	10.81 ± 2.350	0.124	21.92 ± 3.632	0.161
GDX-Vehicle	16.94 ± 3.321	–	9.62 ± 1.923	–	20.29 ± 4.123	–
GDX-10 µg/0.5 µl T	28.36 ± 4.350	0.000***	11.92 ± 3.065	0.001**	28.42 ± 5.162	0.000***
GDX-40 µg/0.5 µl T	33.18 ± 4.496	0.000***	14.85 ± 3.016	0.000***	30.74 ± 4.839	0.000***
GDX-120 µg/0.5 µl T	28.34 ± 5.723	0.000***	12.16 ± 3.928	0.001**	24.75 ± 4.016	0.000***

** P < 0.01; *** P < 0.001 different from the GDX-vehicle group

Treatment with a low dose of testosterone (10 µg/0.5 µl) increased the number of androgen receptor-ir neurons in all regions of GDX-male rat hippocampus. The most significant increase observed in the DG area by the administration of low dose of testosterone (P < 0.001, at 10 µg/0.5 µl, Table 1).

Also, the 40 µg/0.5 µl dose of testosterone, significantly increased the number of androgen receptor-ir neurons in all areas of GDX-male rat hippocampus (P < 0.001).

A significant increase in the number of androgen receptor-ir neurons observed at treatment with a high dose of testosterone (120 µg/0.5 µl) in CA1 (P < 0.001), CA_3_ (P < 0.01) and DG (P < 0.001) areas of the GDX-male rat hippocampus.

## Discussion

The present study was carried out to explore the effects of gonadectomy and testosterone therapy on spatial memory and the density of androgen receptor-ir neurons in rats’ hippocampus. We found that bilaterally pre-training microinjection of 10, 40 and 120 µg/0.5 µl testosterone into subventricular zone of GDX-male rats’ enhanced spatial memory in the 5-day protocol of Morris water maze task.

In some other studies, a lack of difference in the acquisition or retention of reference memory (memory for information consistent across trials or sessions) between GDX and intact males has been reported in rodents responding on both the visible and hidden platform versions of the Morris water maze task [52–54].

However, in this study, we found that GDX-vehicle group has further latency to find the hidden platform in the Morris water maze in comparison to intact-no T group. In the Morris water maze, GDX male rats required more time to find the hidden platform (latency period) relative to those that received saline, an indication of compromised spatial memory [55]. Indeed, gonadectomy decreases cognitive performance of male rats [56]. In addition, the exogenous testosterone replacement reversed the cognitive impairments resulting from gonadectomy [52, 57–60]. But other studies reported that androgens such as testosterone enhanced spatial memory [15, 61]. Also, some studies showed that orchidectomized rats committed a greater number of errors than rats subjected to sham surgeries during the training phases of several different versions of the radial-arm maze task [53, 62].

Results from the other studies had indicated a beneficial role for testosterone in male rats on a variety of spatial tasks [52, 55, 60, 62–64] and it may have beneficial effect in ameliorating memory impairments of senile patients suffering from AD. Further clinical studies should be carried out to prove the possible useful effect of testosterone as an adjuvant therapy in AD [65]. In agreement with these studies, our results indicated that testosterone improves spatial memory impairment induced by gonadectomy and intermediate dose of testosterone had been most effective in this case. Whereas studies employing the Morris water maze to test spatial reference memory have shown no effect of testosterone on performance [52, 53, 66, 67].

Testosterone can bind directly to androgen receptors or can be aromatized to estradiol, acting on nuclear estrogen receptors, ERα and ERβ, or reduced to dihydrotestosterone, acting on androgen receptors [68, 69].

In the present study, investigation of the hippocampus showed that, gonadectomy decreased the density of androgen receptor-ir neurons in all subfields of hippocampus and testosterone therapy could increase density of these neurons in all areas of the hippocampus. In male rats and mice, androgens, more so than estradiol, modulate the expression of a variety of morphological and neurochemical endpoints in the hippocampus believed to underlie spatial cognition [70].

It is noteworthy that previous light microscopic studies have shown that androgen receptor mRNA, immunoreactivity and binding are present in pyramidal cell nuclei but not granule cells [5, 41, 71, 72]. However, androgen receptor-ir is present in disperse, punctuate processes that are most dense in the pyramidal cell layer and diffusely distributed in the mossy fiber pathway [73]. Electron microscopic analysis revealed androgen receptor-ir at several extranuclear sites in the DG. Androgen receptor labeling has been found in dendritic spines, many arising from granule cell dendrites. Androgen receptor is affiliated with clusters of small, synaptic vesicles within preterminal axons and axon terminals, the majority of these being in the central hilus. Androgen receptor-ir preterminal axons are most prominent in the CA3 stratum lucidum. Androgen receptor-labeled terminals exclusively form asymmetric synapses. Throughout the DG, androgen receptor-ir has also been detected in astrocytic profiles; many of them apposing terminals that synapse on unlabeled dendritic spines or forming gap junctions with other androgen receptor-positive or unlabeled astrocytes [73]. Together, these results suggest that androgen receptors may serve as both a genomic and non-genomic transducer of androgen action in the DG [74]. Our results are in agreement with these reports that indicated in addition to the CA1 area, the androgen receptor-ir neurons also identified in CA3 and DG regions of the hippocampus.

The expression of androgen receptors in the hippocampus, particularly in area CA1, is significantly reduced following the removal of testicular hormones [5, 75]. Our results showed that in addition to the CA1 area, the number of androgen receptor-ir neurons also reduced in CA3 and DG regions of the hippocampus following the gonadectomy. In agreement with our data, other studies demonstrated that orchidectomy-induced impairments in spatial memory result from a reduction in the expression of androgen receptors in the hippocampus [70]. It is also possible that hippocampal new neuron production mediates androgen effects on spatial memory [76–78]. In mice, castration disrupts spatial memory and reduces the immature neuron number, but there is no strong link between these effects [79].

In this study, testosterone (40 µg/0.5 µl) could decrease latencies to find the invisible platform, and also it increased significantly the number of androgen receptor-ir neurons in the CA1 area of the hippocampus. In agreement with our findings, other studies demonstrated that a decrease in the level of testosterone had deleterious histological effects in the rat hippocampus. Testosterone replacement ameliorated these histological changes after orchiectomy [80].

## Conclusion

To summarize, it can be concluded that gonadectomy (GDX-vehicle group) caused memory impairments and it can decrease the density of androgen receptor-ir neurons in CA1, CA3, DG areas of the hippocampus. Also we concluded that testosterone can compensate memory failure in gonadectomized rats, and also the density of androgen receptor-ir neurons in rats’ hippocampus is dependent to testosterone. But this relation was not linear.
